# Laser power meters as an X-ray power diagnostic for LCLS-II

**DOI:** 10.1107/S1600577517014096

**Published:** 2018-01-01

**Authors:** Philip Heimann, Stefan Moeller, Sergio Carbajo, Sanghoon Song, Georgi Dakovski, Dennis Nordlund, David Fritz

**Affiliations:** a Linac Coherent Light Source, SLAC National Accelerator Laboratory, 2575 Sand Hill Road, Menlo Park, CA 94025, USA; b Stanford Synchrotron Radiation Laboratory, SLAC National Accelerator Laboratory, 2575 Sand Hill Road, Menlo Park, CA 94025, USA

**Keywords:** power meter, diagnostics, X-ray free electron laser

## Abstract

For the LCLS-II X-ray instruments, laser power meters are being developed as compact X-ray power diagnostics to operate at soft and tender X-ray photon energies. These diagnostics can be installed at various locations along an X-ray free-electron laser (FEL) beamline in order to monitor the transmission of X-ray optics along the beam path. In addition, the power meters will be used to determine the absolute X-ray power at the endstations. Here, thermopile power meters, which measure average power, and have been chosen primarily for their compatibility with the high repetition rates at LCLS-II, are evaluated. A number of characteristics in the soft X-ray range are presented including linearity, calibrations conducted with a photodiode and a gas monitor detector as well as ultra-high-vacuum compatibility tests using residual gas analysis. The application of these power meters for LCLS-II and other X-ray FEL sources is discussed.

## Abstract 2

Laser power meters are being developed as a compact X-ray power diagnostic for LCLS-II and are characterized for their responsivity, linearity and vacuum compatibility. The power meters are calibrated against X-ray photodiodes and a gas monitor detector.

## 1. Introduction   

The LCLS-II project is constructing a 4 GeV superconducting accelerator for the production of X-ray free-electron laser (FEL) radiation. LCLS-II will generate X-rays at repetition rates up to 0.93 MHz and cover a photon energy range from 250 to 5000 eV. The average power is expected to exceed 200 W over most of the energy range. LCLS-II operations are scheduled to begin in 2020.

At X-ray FELs, measuring absolute intensities is still a challenge because of the high peak power. For the X-ray transport, it is important to monitor the transmission of the X-ray beamline to capture intensity losses, for example due to contaminant deposition on mirror coatings. At the endstations, measured intensities allow experimenters to calculate expected count rates and to design experimental configurations effectively. For beamline characterization, the X-ray beam may be blocked, and a slow measurement averaging over a number of pulses is sufficient. Applications such as nonlinear X-ray spectroscopy require a comprehensive characterization of the X-ray pulse at the sample, including its power density.

A number of intensity diagnostics have been developed for X-ray FELs such as the gas monitor detector (GMD), the intensity position monitor (IPM) and a room-temperature calorimeter. The GMD uses the photoionization from rare gas atoms (Tiedtke *et al.*, 2014 ▸). It is non-invasive because of the low gas density, and provides absolute intensities from a calibration at the Radiometry laboratory of the PTB at BESSY. However, a considerable length along the X-ray path is needed for the GMD and the associated differential pumping. In addition, the GMD requires fairly complex controls. The IPM detects back-scattered X-rays from thin, partially transmissive silicon nitride or diamond foils (Feng *et al.*, 2011 ▸; Tono *et al.*, 2011 ▸). With a target thickness chosen according to the photon energy and photoabsorption cross section, the transmission of the IPM can be high, and its responsivity can be calibrated (Kato *et al.*, 2012 ▸). But, at high average power, the cooling of the thin targets is challenging. The room-temperature calorimeter is based on the equivalence of electrical and radiant heating (Tanaka *et al.*, 2015 ▸). The calorimeter achieves a high accuracy for power measurements on the milliwatt level.

Optical laser beam diagnostics are well established today and multiple commercially available solutions exist. In general, there are two types of optical intensity diagnostics: one measures power and the other measures energy. In the power detectors, the temperature difference is detected between an absorber and a heat sink. These power detectors have a relatively slow response and provide an average measurement of the radiation power. In the energy detectors, a pyroelectric material is commonly used to generate an electrical current from a temperature change. The energy detectors can have a fast response and output the pulse-by-pulse energies. For the development of the LCLS-II X-ray power diagnostics, the optical power detectors were chosen because their average response is compatible with any repetition rate. Since the power meters intercept the beam, normalization must be provided by a separate diagnostic. The power meters can provide absolute calibration of other non-invasive pulse-by-pulse energy diagnostics.

**Figure 1:**
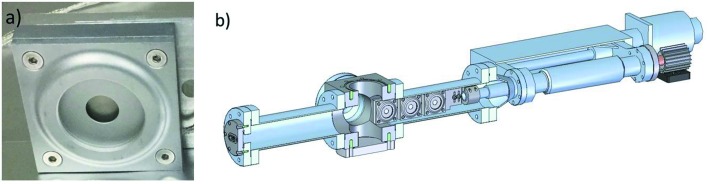
(*a*) Photograph of a Gentec-EO model UP10P-2S-5-TE-D0 power meter. (*b*) Engineering model of the experimental setup.

A thermopile power meter, Gentec-EO model UP10P-2S-5-TE-D0, was selected. A photograph of one of the Gentec power meters is shown in Fig. 1(*a*) ▸. Three power meters (PM1, PM2 and PM3) were procured to allow comparisons between the detectors. This power meter has an aluminium absorber with an unmodified surface and an effective aperture of 10 mm, which is larger than the unfocused X-ray beam diameter at the LCLS Soft X-ray Research (SXR) instrument (2.6 mm FWHM at 800 eV). The dimensions are compact with a total height and width of 46 mm and a 13 mm depth. The power meter signal was conditioned by an amplifier, Gentec-EO model PCB, providing a 0–10 V output. The nominal maximum absorbed power is 2 W, but this limit can be increased based on the cooling method.

## 2. Characterization at the LCLS SXR Instrument   

The power meters were installed at the LCLS SXR Instrument (Dakovski *et al.*, 2015 ▸) between the GMD (Tiedtke *et al.*, 2014 ▸) and the S2B and S2C radiation stoppers. The engineering model of the experimental setup is displayed in Fig. 1(*b*) ▸. The three power meters were mounted on a copper rod, and a fan outside the vacuum providing cooling. This cooling is considered sufficient because the LCLS average power is less than 1 W. A motorized linear motion feedthrough is used to translate the power meters into the X-ray beam or alternatively to let the beam pass through. The SXR Instrument was operated in non-monochromatic mode with the X-ray beam impinging on an unruled portion of the grating.

In Table 1 ▸, the responsivity of the power meters at 1510 eV photon energy is shown. A background voltage, the signal with the power meter out of the X-ray beam, has been subtracted followed by normalization to the pulse energy measured by the gas detectors in the front-end enclosure (Hau-Riege *et al.*, 2010 ▸). The gas detectors are calibrated by electron beam energy-loss measurements (Emma *et al.*, 2010 ▸), but the pulse energy determined by the gas detectors corresponds to the X-ray beam directly from the LCLS source. Between the front-end enclosure, where the gas detectors reside, and the power meters are four mirrors and a grating. Thus the measurements were relative and the absolute power calibration will be described in the next section. The measurements were performed over two days and provided promising results. The responsivity of the three power meters was the same within 6% and the repeatability of the power meters was good to within 2%. These results suggest that it would be possible to install uncalibrated power meters at various points along an X-ray FEL beamline to provide meaningful approximate values of the transmission at the different locations. Because the power meter measurement is based on the temperature of the aluminium absorber, it is expected that the response should be insensitive to possible contamination of the absorber surface and should exhibit good long-term stability.

**Figure 2:**
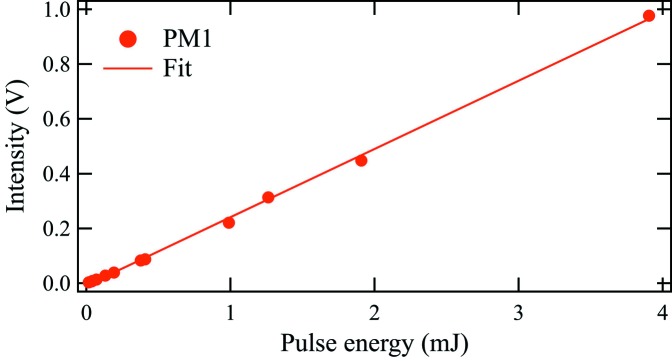
Linearity of PM1 at 1510 eV photon energy.

The linearity of the power meters was tested by varying the X-ray pulse energy using the gas attenuator in the front-end enclosure. Fig. 2 ▸ displays the PM1 signal *versus* the pulse energy derived from the measured value from the upstream gas detector and the calculated gas attenuation. A background power meter signal has again been subtracted. From a comparison of the fitted line and data points, the responsivity is linear to within a standard deviation error of 0.01 V. Measurements were performed with LCLS pulse energies ranging from 16 µJ to 3.9 mJ. The power meters demonstrate a dynamic range of two orders of magnitude. Previous results from the GMD at the SXR Instrument (Moeller *et al.*, 2015 ▸) predict the X-ray pulse energy at that location of the SXR beamline to be 23% of the value in the front-end enclosure. At LCLS, this dynamic range is well matched to the non-monochromatic mode, but the monochromatic mode is at the lower limit of the power meter sensitivity. At LCLS-II, the power will be considerably larger because of the high repetition rate.

**Figure 3:**
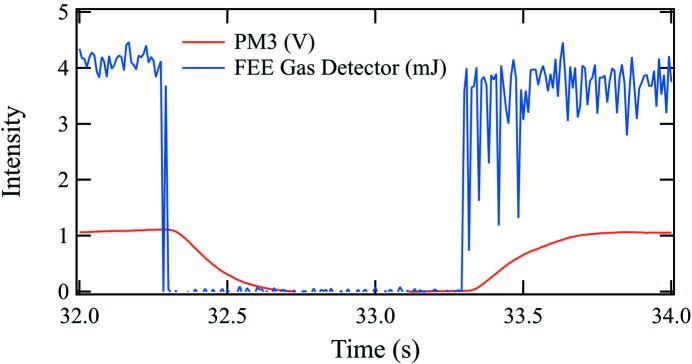
Response time of PM3 for a short period during which the beam was lost.

The response time of the power meters can be observed from periods of time when the beam was temporarily lost, as shown in Fig. 3 ▸. The gas detector observes the energy of each X-ray pulse. The structure in the gas detector curve shows when the LCLS was not stable. The power meter curve is smooth from the averaging over its response time. The time for the PM3 voltage to decrease by 1/e is 0.18 s. Similarly, for the other power meters, the 1/e time was in the range 0.14–0.18 s.

## 3. Calibration of the power meters with a photodiode at SSRL beamline 10-1 and with the gas monitor detector at the LCLS SXR Instrument   

Power meters were further calibrated both with a photodiode at the Stanford Synchrotron Radiation Laboratory (SSRL) beamline 10-1 and with the gas monitor detector at the LCLS SXR Instrument. The calibration was made against a silicon photodiode, Opto Diode model AXUV100, measured with a Keithley current amplifier. Photodiodes are the established detector of choice for measuring absolute intensities of synchrotron radiation (Krumrey & Tegeler, 1990 ▸; Scholze *et al.*, 1996 ▸). Beamline 10-1 has a wiggler X-ray source and a spherical grating monochromator with two gratings, 600 and 1000 lines mm^−1^, covering the photon energy range 200–1400 eV. A titanium filter, 0.5 µm thick, was used to suppress and evaluate the higher-order contributions. The synchrotron radiation power even from a wiggler beamline was relatively low for the power meter sensitivity, and, in order to maximize the power, the entrance and exit slits were set to 200 and 1900 µm, respectively, generating 1–15 mV on the power meters. This signal was of the same magnitude as the power meter background while the background varied over a few 0.1 mV. To provide an accurate background subtraction, the power meter background was sampled ten times before each scan segment over a 100 eV photon energy width.

The following expression was used for the photodiode responsivity (Krumrey & Tegeler, 1990 ▸),
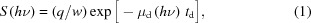
where *q* is the electron charge, *w* is the mean electron–hole creation energy, μ_d_ is the X-ray absorption coefficient and *t*_d_ is the surface dead layer thickness. A value of 3.64 eV is taken for *w* (Scholze *et al.*, 1996 ▸). For the photon energies measured here, the transmission through the effective silicon thickness can be neglected. Scholze *et al.* present a more sophisticated model of the photodiode responsivity; however, the differences between the models is significantly smaller than the experimental uncertainty here. *t*_d_ was characterized by measuring the photodiode current at normal and 30° grazing-incidence angles from 200 to 800 eV photon energy. The two incidence angles result in a factor of two difference in the X-ray path length in the dead layer. A fit of the measured photodiode response gave equivalent thicknesses of 5 nm carbon, 4 nm oxygen and 6 nm silicon dioxide layers. It is considered that carbon and oxygen are contaminants on the silicon dioxide passivating layer.

At the LCLS SXR Instrument, the power meters were installed downstream of the gas monitor detector (Moeller *et al.*, 2015 ▸) without any X-ray mirrors between them. Because of the low GMD operating pressure, its transmission is nearly 1. During the power meter characterization, average pulse energies were determined by the GMD using krypton gas and the calibration procedure for absolute average pulse energies as described by Tiedtke *et al.* (2008 ▸). These results provide a second calibration data set for the power meters.

**Figure 4:**
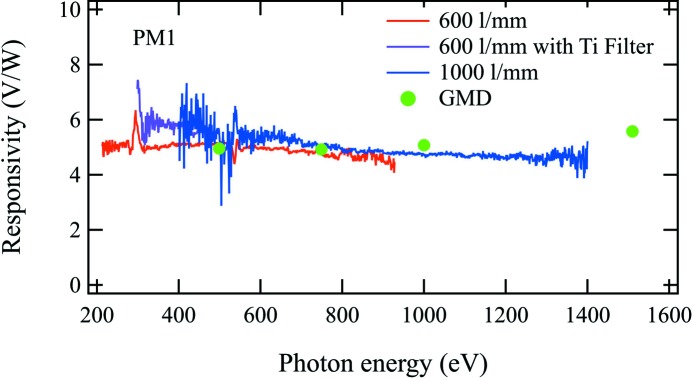
Responsivity of PM1 as a function of photon energy measured with a photodiode at SSRL BL 10-1 and with the GMD at LCLS SXR.

As shown in Fig. 4 ▸, the SSRL photodiode calibration obtained with different gratings and with and without the filter shows the measured PM1 responsivity that varies between 4.6 and 5.4 V W^−1^ in the photon energy range from 200 to 1400 eV. Additional structure is observed at 294 and 538 eV photon energies, which we interpret as resulting from C and O on the photodiode or power meter surfaces. Based on the SSRL photodiode data, a calibration uncertainty of 12% is estimated from the variation of the power meter responsivity with photon energy. From the GMD calibration at photon energies between 500 and 1510 eV, the observed PM1 responsivity is between 4.9 and 5.6 V W^−1^. The uncertainty of the GMD calibration combining the contributions from the GMD (Tiedtke *et al.*, 2014 ▸), the photon energy and the power meters is estimated to be 7%. The power meter calibrations using the photodiode at SSRL and employing the GMD at SXR are in agreement within the experimental uncertainties.

Two possible loss mechanisms that could affect the power meter responsivity are X-ray fluorescence and electrons escaping from the absorber surface. The fluorescence yield for Al *K*α is only 3.9% while the yield for the Al *L* emission is significantly weaker and can be neglected (Krause, 1979 ▸). Note that half of these fluorescent X-rays will be emitted toward the bulk of the absorber and will be reabsorbed while another fraction will be reabsorbed before reaching the surface. The Al *K*α transition is only allowed at photon energies above the Al *K*-edge at 1560 eV. The yield of photoemitted electrons depends on the relative magnitude of the X-ray penetration depth and the electron escape depths. For soft X-rays, the electron yield from aluminium varies from 0.6 to 6% (Henke *et al.*, 1981 ▸) and, as is usually the case for photoemission, the strong majority of the emitted electrons are secondary electrons with low kinetic energy. Consequently, the power lost through electrons is significantly less than the electron yield. Since both the X-ray fluorescence and photoemission mechanisms are inefficient, the responsivity of the power meters is expected to be nearly constant, independent of the photon energy.

## 4. Vacuum qualification   

**Figure 5:**
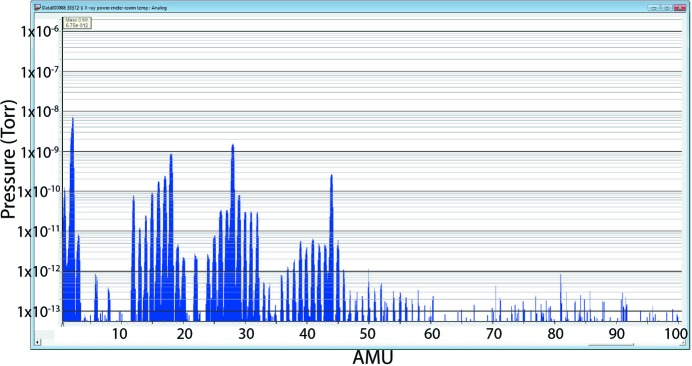
RGA scan of PM3 at room temperature after bakeout.

How the power meters alter the vacuum is a concern for LCLS-II, since the power meters will be installed in beamline locations close to X-ray mirrors. The pressure in the mirror chambers cannot be compromised in order to minimize the contamination of the optical surfaces over time. Residual gas analysis (RGA) scans were performed during and after a bakeout at the maximum allowable temperature of 100°C. Fig. 5 ▸ shows an RGA trace measured at room temperature after a bakeout of 30 h at 100°C. The RGA result meets the LCLS vacuum specification for beamline components, which requires at room temperature that the sum of partial pressures of all peaks above 44 a.m.u. must be less than 1 × 10^−11^ torr and that the maximum single partial pressure above 44 a.m.u. must be less than 5 × 10^−12^ torr. Further discussions will be conducted with the vendor to minimize the use of non-UHV compatible materials.

## 5. Summary   

Measurements with thermopile power meters have been performed at the LCLS SXR Instrument. The responsivity of three power meters was observed to be the same within 6%. The linearity was confirmed over two orders of magnitude in the X-ray power. A calibration of the responsivity of the power meters was carried out against a silicon photodiode at the SSRL synchrotron radiation facility and a gas monitor detector at the SXR Instrument. The two calibrations were in agreement within the uncertainties, and the power meter responsivity showed an expected insensitivity to the photon energy. The vacuum performance of the power meters meets the LCLS ultra-high-vacuum criteria for X-ray transport components. The power meters have already been used to evaluate the transmission change from the *in situ* cleaning of the LCLS AMO Kirkpatrick–Baez mirrors. A power meter with cooling suitable for the powers anticipated at the LCLS-II instruments will be developed. In conclusion, these power meters show great promise as absolute average-power diagnostics for LCLS-II and other X-ray FEL sources with compact dimensions and uncomplicated installation and operation.

## Figures and Tables

**Table 1:** Comparison of the responsivity of the three power meters at 1510 eV photon energy

Date	PM1 (V mJ^−1^)	PM2 (V mJ^−1^)	PM3 (V mJ^−1^)
9 November 2016	0.248	0.271	0.271
16 November 2016	0.253	0.272	0.269
